# The role of Chinese Milk Vetch as cover crop in complex soil nitrogen dynamics in rice rotation system of South China

**DOI:** 10.1038/s41598-018-30239-6

**Published:** 2018-08-13

**Authors:** Zhijian Xie, Chunhuo Zhou, Farooq Shah, Amjad Iqbal, GuoRong Ni

**Affiliations:** 1 College of Land Resource and Environment, Jiangxi Agricultural University, Nanchang Jiangxi, 330045 P.R. China; 2 Department of Agriculture, Abdul Wali Khan University Mardan, 23200 Kyber Pakhtunkhwa, Pakistan

## Abstract

The effect of Chinese Milk Vetch (CMV) residues as a cover crop on the performance of rice plants and nitrogen mobilization and mineralization in paddy soil was evaluated in a pot-culture experiment. Three treatments were included in the trial, i.e. without exogenous-N (Control or CK), urea fertilizer as the sole N-source (N) and urea plus CMV (NM). The results revealed higher amounts of total-N, inorganic-N, acid hydrolysable-N and non-acid hydrolysable-N in the soil under NM, followed by N and CK treatments at tillering, booting and maturity stages of rice. A similar trend was observed for various soil ammonia oxidizing bacteria, aerobic and anaerobic bacteria. Correlation coefficients exhibited a negative correlation of residual exogenous-^15^N with only amino acid nitrogen (*P* < 0.01). Moreover, low abundance of the key functional gene amoA in rice was found in CK treatment. From the results it can be concluded that the nitrogen from organic source can improve the availability of total-N, inorganic-N and NH_4_ in the soil, especially at the later stages of rice growth.

## Introduction

Rice is a staple food in most parts of China as well as many other developed and developing countries^1^. With the soaring trends in the population around the globe, it is necessary to produce more rice in terms of quantity and quality. Such a goal can be achieved through smart cultural practices and with the development of high yielding cultivars. In case of smart cultural practices, proper management of nitrogen in the paddy field of rice can ensure high yield and quality of rice. The importance of nitrogen is certainly due to its structural and functional role in the plant cell, including proteins, enzymes, cell membrane and cell walls. Therefore, an optimum amount of this important nutrient in the soil is one of the prerequisites of healthy growth and development of the plants. Similarly, the management of nitrogen from natural sources will not only boost up the N contents of soil and growing crops, but also plays important role in reducing environmental pollution^2,3^. Previous studies have demonstrated that the yield of a crop can be increased by controlling the nutrient cycling^4–6^, thus the importance of cover crop management cannot be ignored^7–10^. Cover crops can primarily be used to improve the physicochemical properties of the soil, such as recovering collective stability of the soil, supplementing organic matter to the soil, fixing nitrogen and foraging soil nitrogen. Proper management of the cover crop can regulate nutrient cycles efficiently and ensures high crop production. Also, use of cover crop can reduce soil erosion to increase fertility of the soil that can lead to sustainable agriculture^11^.

In the last few decades, the use of leguminous crops (especially vetch) in a rice crop rotation system got greater attention because of its adaptability to cold and wet paddy soil system. However, inappropriate application of green manure to paddy field can lead to N losses in the form of nitrate, which can be detrimental to the environment^12^. Therefore, it is quite necessary to optimize the proper dosage of the milk vetch before application on a larger scale^13^. The optimum dosage of the residues can favorably affect the physicochemical as well as biological properties and could help in attaining high yield^14^. Additionally, the use of cover crop can influence the microbial population in the soil that is mainly associated with N-cycling^15^. These soil microorganisms have the ability to control the availability of the nutrients (including N) to the plants^16^. The microorganisms in the soil, mainly ammonium oxidizing archea (AOA) and ammonium oxidizing bacteria (AOB) can control the nitrogen cycle by using amoA gene through nitrification^17,18^. Both AOA and AOB can live together in the cultivated soil depending on the availability of urea for AOB and manure for AOA^19,20^. Therefore, the present study tried to answer how the returning cover crop can influence the paddy soil dynamics (such as the availability of organic N and inorganic N, % of acid hydrolysable-N and non-acid hydrolysable-N and population dynamics of soil microbes) and improve the quality and yield of rice.

## Materials and Methods

### Experimental soil and crops

A bulk paddy soil (0–20 cm), was collected from Quaternary red soil at Fengcheng, Jiangxi. Soil characteristics was based on the analysis of soil samples taken as cores of known volume (height 5 cm, ∅7.14 cm) from the undisturbed field soil. The soil characteristics were; soil pH 5.41, soil: water ratio was 1: 2.5, total N = 1.35 g kg^−1^ (Kjeldahl method), soil organic carbon = 16.0 g kg^−1^ (the Walkley and Black method), extractable P = 8.52 mg kg^−1^ (the Olsen’s method), available K = 68.0 mg kg^−1^ (with ammonium acetate) and soil bulk density was 1.47 g cm^−3^. The cores were oven-dried and weighed following the procedure as described by Page^21^. The climatic conditions of the experimental region can be characterized as subtropical monsoon, exhibiting heavy rain from March to June and a drought session from September to December. The average annual temperature of the region is 17.8 °C with an average annual rainfall of 1545.9 mm, annual sunshine of 1603.4 h and an average frost-free period of 276 days.

The rice cultivar used in the experiments was Qianyou 1^#^ (*Oryza sativa subsp*. *Indica*. var. Qianyou 1^#^), and the model winter cover crop was Chinese Milk Vetch (CMV, var. Ganzi 1^#^, *Astragalus sinicus* L.).

### Preparation of labeled (^15^N) winter cover crop plants

The seeds of CMV were sterilized with 3% (v/v) NaOCl for 30 min and were thoroughly rinsed with tap water to remove traces of NaOCl. The sterilized seeds were sown without the inoculation of rhizobium in a plastic containers (100 cm × 50 cm × 10 cm) filled with 25 kg of clean quartz sand (Nitrogen-free). The humidity inside the containers was kept constant by placing them in a larger plastic container having 5 cm of water at its base. Each container was then covered with a transparent thin plastic film. During the growth period, a 600–1000x dilution of 50% carbendazim (wettable powder) and 75% thiophanate (wettable powder) were used as a foliar spray against sclerotinia and powdery mildew. Additionally, 0.03% (m/m) of pirimicarb was used as a foliar spray to prevent any leaf miner pest.

CMV seedlings got fertilized once per week with a Hoagland-Arnon nutrient solution (2x dilution) after attaining a height of 3–4 cm and having 2–3 fronds^22^. Nitrogen was also applied as a nutrient in the form of urea-^15^N (99.24 atom%, provided by the Shanghai Research Institute of Chemical Industry) for culturing ^15^N-labeled (^15^NM).

The ^15^NM CMV plants were harvested at full-bloom stage and were washed carefully with the tap water and later rinsed with Milli-Q water (Millipore, USA). The water on the surface of the plants surface were dried with gauze and then transferred to the oven operated at 105 °C. The plants were kept in an oven for 30 minutes to deactivate the enzymes. The plants were taken out and the temperature of the oven was brought to 70 °C. Finally, the plants were oven dried at 70 °C for 24 hours and weighed. The dried and weighed CMV plants were divided into two equal portions. One portion was chopped into pieces of 5 mm, which were stored in a refrigerator at 4 °C for the future experiments, whereas the other portion was ground to fine powder and sieved through a 0.5-mm mesh for analysis. The ^15^N-labeled CMV plants had 3.22 g kg^−1^ N. The total ^15^N abundance of the labeled CMV plants was 42.27% that was measured with the isotope mass spectrometer (Thermo-Fisher Delta V Advantage IRMS, USA) by the method of Buresh *et al*.^23^.

### Experimental design and crop management

A pot experiment was conducted by applying cross ^15^N isotope labeled technique with urea and CMV as nitrogen sources in a greenhouse. The size of stainless steel container was 40 cm × 40 cm × 30 cm, which was filled with 47 kg of the air-dried soil. Three treatments were included in the trial, which were replicated three times.

1. Without exogenous N (Control or CK)
2. Urea fertilizer as the sole N-source (N)
3. Urea plus CMV materials (NM)

The dry CMVs (at a rate of 0.71 g kg^−1^) were added to the soil as a basal dose 30-days prior to rice transplantation. The total amount of nitrogen applied in the N and NM treatments was kept same. The mixtures of N (0.12 g kg^−1^), P_2_O_5_ (0.05 g kg^−1^) and K_2_O (0.1 g kg^−1^) were applied as a basal dose a day before rice transplantation. The sterilized rice seeds were sown in pottery pots (20 cm × 20 cm) that were filled with 5 kg air-dried soil. When the rice seedlings reached a height of 7–8 cm (having 3–4 fronds), they were transferred to a stainless steel container having 9 caves (2 seedlings per cave).

### Soil sampling procedure

For all four treatments, the topsoil samples (0–20 cm) with a soil corer of ∅3 cm were taken at tillering, booting and maturing stages of rice plants. From the pots of each treatment, five cores of soil were randomly collected and mixed thoroughly inside a plastic bucket to form individual bulked samples. After removing visible roots and stones, the soil samples were divided into two parts: one was freeze-dried immediately and stored at −70 °C for phospholipid fatty acid (PLFA) analysis and remaining soils were air-dried and sieved (<0.149 mm) prior to chemical analysis.

### Analytical methods

#### Soil organic-N fractions analysis and 15N enrichment measurement

According to the method described by Stevenson^24^, three replicates of each soil sample (<0.149 mm) containing 10 mg of nitrogen were fractionated by a step-wise acid hydrolysis. Organic forms of nitrogen were measured in the hydrolysate prepared by refluxing the soil sample with 6 M HCl at 120 °C for 12 h, using the oil bath. Total hydrolysable-N was determined by steam distillation with 10 M NaOH after Kjeldahl digestion of the acid hydrolysate. Hydrolysable ammonium was measured by steam distillation with 3.5% (w/v) MgO. Amino sugar-N was calculated as a difference of N obtained by steam distillation of the hydrolysate with phosphate-borate buffer at pH 11.2 and the N of hydrolysable ammonium. Amino acid-N (α-amino acid-N) was determined by steam distillation of an aliquot of the hydrolysate with phosphate–borate buffer and 5 M NaOH after treating with 0.5 M NaOH at 100 °C to decompose hexosamines and removing NH_3_-N. Ninhydrin powder was added to convert the amino-N to NH_3_-N. The amounts of hydrolysable unidentified-N (HUN) and non-acid hydrolysable-N (NHAN) were calculated by the following formula (1) and (2), respectively.

1 $$HUN=total\,hydrolysableN-(ammonium\,N+amino\,acid\,N+amino\,sugar\,N)$$

2 $$NHAN=total\,N-acid\,hydrlysable\,N$$

The resulting ammonium sulphate solutions were then acidified with 5 mL of 0.005 M H_2_SO_4_ and oven dried at 80 °C near a vial of 18 M H_2_SO_4_ to prevent possible contamination by atmospheric ammonia. ^15^N abundance was determined with Isoprime100 mass spectrometer (Germany) operating in a continuous flow with a CN elemental analyser (Elementar of Vario Isotope Cube, Germany). Accordingly, the contributions of exogenous-^15^N to each fraction of soil organic-N were calculated by the following formula (3)

3 $${N}_{dff}=\frac{{R}_{e}}{{R}_{f}}\times 100 \% $$

where *N*_*dff*_ was the proportion of exogenous-^15^N to each fractions of soil organic-N content, *R*_*e*_ was the ^15^N atom% in different fractions of soil organic-N and *R*_*f*_ represented the ^15^N atom% of exogenous-N (^15^NU or ^15^NM).

#### Soil microbial communities and populations

The soil microbial community was characterized using phospholipid fatty acid (PLFA) analysis. The PLFA was extracted from the soil using the procedure described by Wu *et al*.^25^. Total lipids were extracted from 8 g of soil sample using potassium phosphate, chloroform and methanol buffer. Phospholipids were fractionated from neutral and glycolipids on a silica column. After mild alkaline methanolysis for the production of fatty acid methyl esters (FAMEs), samples were dissolved in hexane and analyzed in an Agilent 6890 N gas chromatograph with Agilent 19091B-102 (25.0 m × 200 µm × 0.33 µm) capillary column. Hydrogen was use as a carrier gas and the fatty acid 19:0 was added as an internal standard before methylation. The fatty acid methyl esters were identified automatically by the MIDI peak identification software (version 4.5; MIDI Inc. Newark, DE)^26^. The following fatty acid nomenclature was used: total number of carbon atoms: number of double bonds, followed by the position of the double bond from the methyl end of the molecule. Cis and trans configurations are indicated by c and t, respectively. Anteiso- and iso-branching are designated by the prefix a or I. Methyl group on the 10^th^ carbon atom from the carboxyl end of the molecule was shown as 10Me^27^. Total amounts of the different PLFA biomarkers were used to represent the different groups of soil micro-organisms. The sum of the following PLFA biomarkers were considered to represent bacterial origin (gram-positive bacteria by i14:0, i15:0, a15:0, i16:0, a16:0, i17:0, a17:0, gram-negative bacteria by 16:1v9c, cy17:0, 18:1v5c, 18:1v7c, cy19:0, bacteria were represented by the sum of the two)^28^. Biomarkers 18:3v6c, 18:1v9c and 16:1v5c were used for fungal PLFA, and 10Me16:0, 10Me17:0 and 10Me18:0 were used for actinomycetes PLFA^29^. All of the PLFA biomarkers indicated above were considered to be the representative of total PLFA of the soil microbial community.

### Statistical analysis

All data represent arithmetic means ± standard deviation (SD) of three replicated analysis and were statistically analysed by one-way ANOVA and significant differences were distinguished by the Tukey-HSD test at *P* < 0.05 levels using SAS statistical package (Version 9.1, Cary, USA).

Correlation coefficients between different soil organic-^15^N fractions and residual-^15^N in paddy soil. Stepwise regression of soil residual-^15^N with soil organic-^15^N fractions at different growing stages of rice plants were done using SAS statistical package (Version 9.1). To have a deeper insight into, the direct and indirect effects of various soil organic-^15^N fractions on dependent variables (soil residual-^15^N), the path coefficient analysis was worked out. Path coefficient analysis splits up the correlation coefficients between each pair of dependent variables and independent variables into a direct effect (path coefficient) and as indirect effects (path coefficient × correlation coefficient). Thus, the correlation coefficients between dependent variables and independent variables, which are of utmost importance, are the summation of direct and indirect effects. Path coefficient analysis was done as per the method suggested by Dewey and Lu^30^. Sigmaplot 10.0 and MS excel were used to generate the graphs.

### Ethics approval and consent to participate

The current study doesn’t involve any human, animal or endangered species.

### Availability of data and material

All the data are included in the manuscript

## Results

### Effect of CMV on total-N and inorganic-N

**Figure 1:**
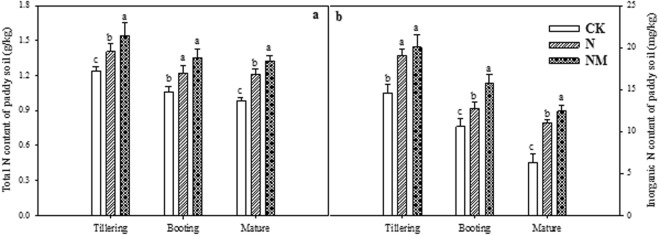
Effects of returning cover crop on total N (**a**) and inorganic N (**b**) content of paddy soil at different growing stages of rice plant. Bars represented by different letters are significantly at *P* < 0.05, whereas error bars represent ±SE of triplicated data.

Data regarding the effect of returning cover crop on the total-N and inorganic-N contents of paddy soil at different growth stages revealed almost similar and consistent trends (Fig. 1a,b). Both total-N and inorganic-N were significantly reduced under NM compared to the control (CK) and N treatments irrespective of the growth stages. Furthermore, as the growth stages progressed, both total-N and inorganic-N contents were decreased in all treatments (Fig. 1a,b).

### Effect of CMV on NO_3_-N and NH_4_^+^-N

**Figure 2:**
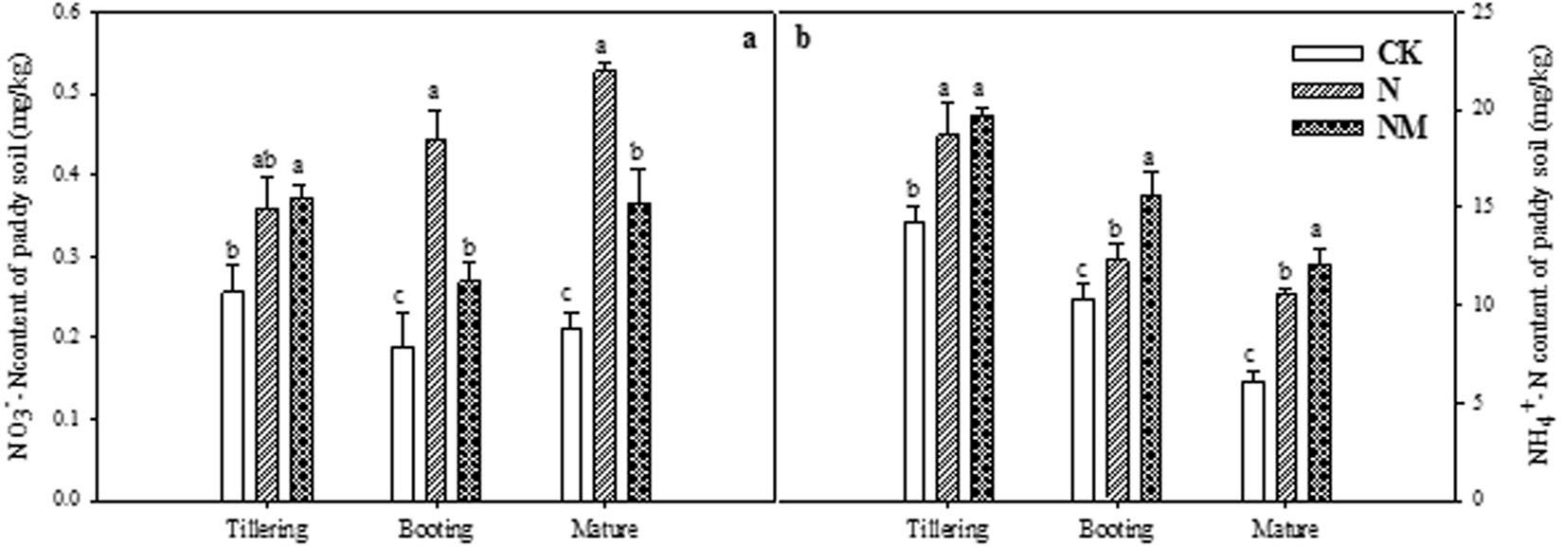
Effects of returning cover crop on NO_3_^−^N (**a**) and NH_4_^+^-N (**b**) content of paddy soil at different growing stages. Bars represented by different letters are significantly at *P* < 0.05, whereas error bars represent ±SE of triplicated data.

The return of cover crop has significantly increased the NO_3_-N content of the paddy soil at different growth stages in both N and NM treatments (Fig. 2a). Moreover, a comparison between N and NM depicts that N treatment significantly increased the NO_3_-N content at booting and maturity in contrast to CK. Likewise, both N and NM treatments also increased the NH_4_^+^-N content at all three growth stages compared with CK (Fig. 2b). The difference between N and NM treatments at tillering was not significant, while at booting and maturity stages, NM treatment exhibited higher NH_4_^+^-N content than N treatment. The NH_4_^+^-N content decreased as the growth stage progressed as maximum value was observed at tillering, whereas the minimum one was recorded at maturity stage (Fig. 2b).

### Effect of different treatments on abundance of amoA gene expression

**Figure 3:**
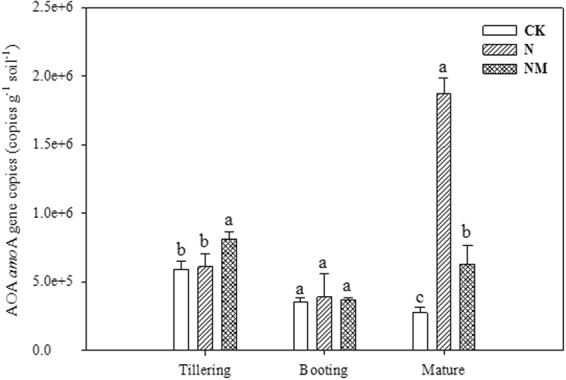
Effects of returning cover crop on the ammonia-oxidizing *Archaea* (*amo*A) gene copies at different growing stages of rice plant. Bars represented by different letters are significantly at *P* < 0.05, whereas error bars represent ±SE of triplicated data.

The results regarding the copies of ammonia-oxidizing archaea gene (amoA) at different growth stages demonstrated high variability (Fig. 3). At tillering stage, NM treatment resulted in significantly higher copies of the amoA gene as compared to CK and N treatments. However, the copies of amoA gene at the booting stage have been recorded in all tested treatments. At the maturity stage the N treatment exhibited higher copies of the amoA gene in comparison to CK and NM treatments. On an overall basis, the copies of amoA gene in case of CK treatment have been the least among all three treatments (Fig. 3).

### Proportion of acid hydrolysable-N and acid non-hydrolysable-N to total-N

**Figure 4:**
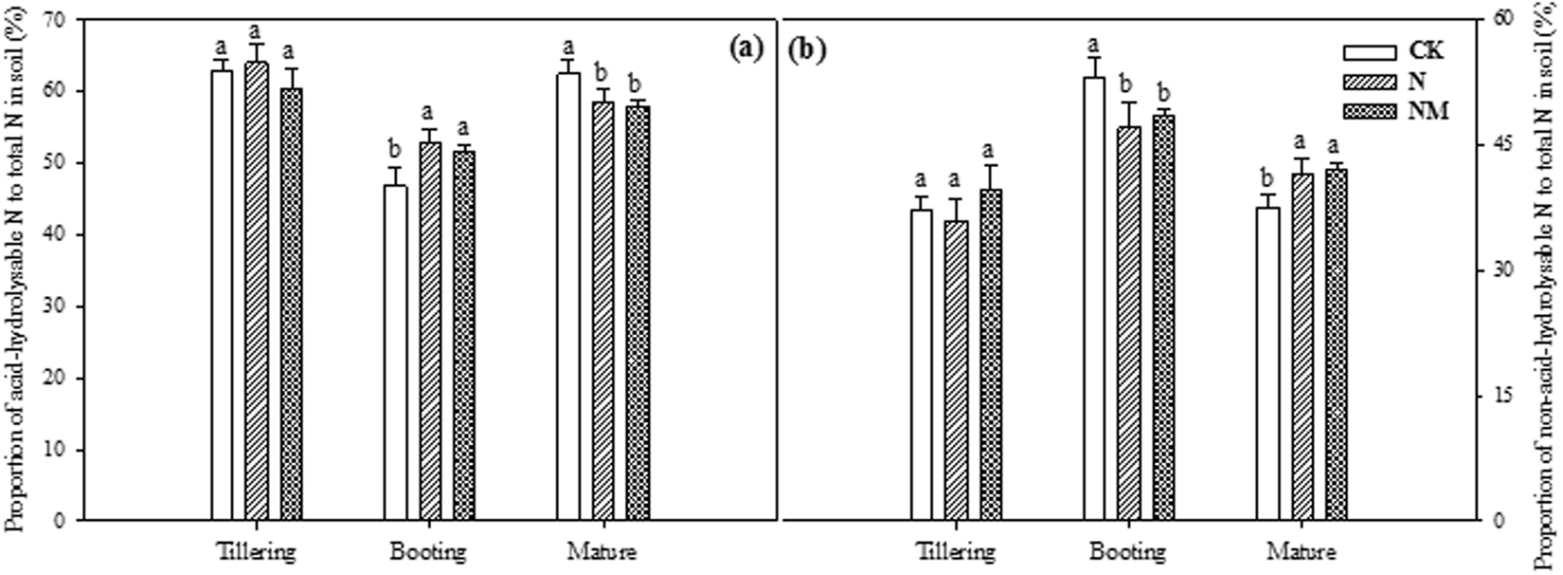
Effects of returning cover crop to paddy field on the proportion of acid hydrolysable-N and non-acid hydrolysable-N to total-N in soil at different growing stages of rice plant. The (**a**) represents acid hydrolysable-N; (**b**) represents non-acid hydrolysable-N in soil at different growing stages of rice plant. Bars represented by different letters are significantly at *P* < 0.05, whereas error bars represent ±SE of triplicated data.

The proportion of acid hydrolysable-N to total-N was higher at tillering stage, which decreased roughly by 10% at booting stage and then increased at maturity stage in all treatments (Fig. 4a). Inconsistencies concerning the proportion of hydrolysable-N to total-N have been noticed throughout the growth period in all three treatments. CK treatments exhibited minimum value at booting stage as compared to NM and N treatments. At the maturity the trend has been reversed to some extent as a maximum proportion of hydrolysable-N to total-N have seen under control treatment (Fig. 4a). Understandably, a completely inverse trend has been noticed for the proportion of non-hydrolysable-N to total-N, where the proportion was higher at tillering stage rather booting and maturity stage (Fig. 4b).

### Effect of CMV on soil microbes

The number of aerobic ammonia oxidizing bacteria (AOB) has been higher in N treatment at both booting and maturity stage, while it has been significantly lower at all stages in CK treatment. The number of aerobic AOB for NM treatment is comparable to those of N treatment (Table 1). A very similar trend has been noticed for the Anaerobic AOB as affected by returning cover crop CMV. The number of anaerobic AOB has been higher in N treatment at booting stage (17 × 10^7^ CFU g^−1^) as compared to the growth stages of rice plant. On an overall basis, the N treatment performed well considering the total AOB (27.3 × 10^7^ CFU g^−1^) among the tested treatments (Table 1).

**Table 1:** Effects of returning cover crop on the ammonia-oxidizing bacteria (AOB) at different growing stages of rice plant.

Treatment	Aerobic AOB (CFU 10^7^/g)	Anaerobic AOB (CFU 10^7^/g)	Total AOB (CFU 10^7^/g)
**Tillering stage**			
CK	2.10 ± 0.11b	1.48 ± 0.33c	3.59 ± 0.82b
N	8.90 ± 0.64a	11.6 ± 1.08b	20.5 ± 1.11a
NM	8.37 ± 0.52a	13.0 ± 0.74a	21.4 ± 2.35a
**Booting stage**			
CK	2.33 ± 0.37b	3.03 ± 0.53c	5.36 ± 0.88c
N	10.3 ± 0.41a	17.0 ± 10.5a	27.3 ± 1.07a
NM	10.1 ± 0.78a	13.1 ± 1.63b	23.2 ± 1.72b
**Mature stage**			
CK	1.26 ± 0.21c	0.49 ± 0.08b	1.75 ± 0.18c
N	10.3 ± 0.60a	6.12 ± 0.79a	16.5 ± 1.91a
NM	6.51 ± 1.07b	5.90 ± 0.87a	12.4 ± 0.94b

Values followed by different letters in a column are significantly different at *P* < 0.05. Each column consists of means of triplicated data with ± S.E of means.

The number of microorganisms from various species in the soil at maturity stage demonstrated a higher number of aerobic bacteria (5 × 10^8^ CFU g^−1^) and anaerobic bacteria (5.09 × 10^8^ CFU g^−1^) in NM compared to the CK treatment. Furthermore, the total number of recorded bacteria in CK treatment has been far lower (2.54 × 10^8^ CFU g^−1^) than the NM treatment (10.1 × 10^8^ CFU g^−1^) and N treatment (7.39 × 10^8^ CFU g^−1^). Likewise, the number of actinomycetes has been higher (14.4 × 10^6^ CFU g^−1^) in NM treated pots. The effect of returning crop on the number of fungi and azotobacter at the maturity stage of the rice plant has been quite interesting. In both cases the total number of fungi (1.63 × 10^5^ CFU g^−1^) and azotobacter (26.8 × 10^5^ CFU g^−1^) has a higher number in the NM treatment (Table 2).

**Table 2:** Effects of returning cover crop on the quantity of microbes in paddy soil at maturing stage of rice plant.

Treatment	Anaerobic	Aerobic	Total	Anaerobic	Aerobic	Total
**Bacteria (CFU 10** ^**8**^ **/g)**				**Actinomycetes (CFU 10** ^**6**^ **/g)**		
CK	0.96 ± 0.02c	1.57 ± 0.12c	2.54 ± 0.11c	4.52 ± 1.56b	5.77 ± 1.01b	10.3 ± 0.57c
N	4.74 ± 0.14b	2.64 ± 0.07b	7.39 ± 0.63b	6.06 ± 0.89a	6.16 ± 0.98b	12.2 ± 0.49b
NM	5.00 ± 0.35a	5.09 ± 1.43a	10.1 ± 1.08a	6.12 ± 1.07a	8.28 ± 1.74a	14.4 ± 1.05a
**Fungi (CFU 10** ^**5**^ **/g)**				**Azotobcter (CFU 10** ^**7**^ **/g)**		
CK	0.46 ± 0.03c	0.53 ± 0.02b	0.99 ± 0.03c	4.84 ± 0.55c	12.5 ± 2.46b	17.3 ± 1.26b
N	0.71 ± 0.07b	0.72 ± 0.03a	1.43 ± 0.55b	8.93 ± 0.87b	17.2 ± 1.75a	26.1 ± 0.79a
NM	0.95 ± 0.04a	0.68 ± 0.05a	1.63 ± 0.37a	10.1 ± 0.46a	16.7 ± 1.03a	26.8 ± 1.05a

Values followed by different letters in a column are significantly different at *P* < 0.05. Each column consists of means of triplicated data with ± S.E of means.

### Effect of CMV on dry matter and nitrogen nutrients of rice plants

The accumulation of dry matter and nitrogen nutrients has been better in NM treatment at all tested stages of rice plants (Table 3). At the tillering stage the accumulation of dry matter has been very low that increased approx. 17 times at booting stage and 25 times at maturity in NM treated rice plants. A very similar trend has been noticed for both CK and N treatments concerning the accumulation of dry matter at various growth stages of rice plant. Upon the maturity, the accumulation of dry matter in unfilled grain has been higher in N treated plants than the NM treated plants. However, NM treated rice plants have secured higher total dry matter at maturity. Similarly, the plants from the NM treatment have more nitrogen nutrients at all stages of their growth except for unfilled grain at maturity. The nitrogen nutrients of the unfilled grain of rice plants from N treatment have been 0.27 g pot^−1^, followed by NM treatment (0.23 ± 0.04 g pot^−1^). Whereas the nitrogen nutrients of the unfilled grain of rice plants from CK treatment (0.09 ± 0.01) have been significantly lower (Table 3).

**Table 3:** Effects of returning cover crop on dry matter and N-nutrient accumulation in above-ground rice plants at different growing stages (g pot^−1^).

Treatment	Tillering	Booting	Mature			
			Straw	Filled-grain	Unfilled-grain	Total
**Dry matter**						
CK	10.7 ± 0.42b	94.7 ± 5.41c	103.9 ± 2.25b	91.9 ± 8.76c	20.1 ± 1.43c	215.9 ± 7.11c
N	12.9 ± 0.62a	180.3 ± 13.5b	158.7 ± 3.73a	141.2 ± 5.71b	33.4 ± 1.40a	333.3 ± 9.02b
NM	13.1 ± 0.39a	220.5 ± 7.14a	163.4 ± 4.18a	159.4 ± 4.42a	30.6 ± 1.04b	353.4 ± 4.39a
**Nitrogen nutrient**						
CK	0.12 ± 0.01b	0.88 ± 0.06c	0.57 ± 0.02c	1.26 ± 0.11c	0.09 ± 0.01c	1.92 ± 0.58c
N	0.50 ± 0.02a	2.79 ± 0.11b	1.14 ± 0.03b	2.75 ± 0.17b	0.27 ± 0.02a	4.16 ± 0.16b
NM	0.51 ± 0.02a	3.82 ± 0.18a	1.24 ± 0.10a	3.20 ± 0.08a	0.23 ± 0.04b	4.67 ± 0.49a

Values followed by different letters in a column are significantly different at *P* < 0.05. Each column consists of means of triplicated data with ± S.E of means.

### Effect of CMV on acid hydrolysable-N and non-acid hydrolysable-N

The effect of returning cover crop to paddy field on the proportion of acid hydrolysable-N and non-acid hydrolysable-N is given in Table 4. The results designated that both the acid hydrolysable-N and non-acid hydrolysable-N has been maximized in the soil at tillering stage in all tested treatment. A dip in the soil nitrogen has been noted at the booting stage that mounted at maturity irrespective of the tested treatments. By comparing the amount of nitrogen in the soil from various treatments, NM performed well. NM treated plots had significantly greater values of nitrogen in all forms, with the exception of acid-hydrolysable unidentified-N (AHUN). The value of AHUN has been higher in N treatment, where the CK treatment has lower AHUN. On the contrary, for other acid hydrolysable-N sources no dip and rise have been seen for AHUN at booting and maturity stages. An inverse trend to acid hydrolysable-N has been observed for the non-acid hydrolysable-N (Table 4).

**Table 4:** Effects of returning cover crop on different organic-N compositions in paddy soil at different growing stages of rice plant (mg kg^−1^).

Treatment	Acid-hydrolysable N					Non-acid hydrolysable N
	AAN	ASN	AHAN	AHUN	Total	
**Tillering stage**						
CK	363.8 ± 12.0b	27.7 ± 3.31c	228.9 ± 6.97b	154.8 ± 10.2b	775.3 ± 34.5b	460.6 ± 10.4c
N	412.5 ± 13.7a	37.0 ± 2.02b	263.3 ± 11.5a	185.7 ± 11.2a	898.5 ± 26.7a	505.1 ± 7.65b
NM	438.8 ± 15.8a	46.8 ± 3.91a	279.1 ± 6.52a	164.7 ± 9.45b	929.4 ± 30.9a	614.2 ± 11.6a
**Booting stage**						
CK	213.4 ± 12.4c	13.7 ± 1.56c	182.8 ± 4.56b	86.4 ± 4.90b	516.2 ± 20.1c	564.6 ± 14.4b
N	322.5 ± 12.0b	22.7 ± 2.51b	203.7 ± 7.30a	97.8 ± 7.81a	646.8 ± 26.7b	576.1 ± 10.5b
NM	370.2 ± 27.6a	29.1 ± 3.15a	211.7 ± 6.04a	89.3 ± 6.05b	699.6 ± 30.9a	656.3 ± 8.35a
**Mature stage**						
CK	305.9 ± 8.89c	22.6 ± 3.43c	221.6 ± 8.68b	64.7 ± 5.66c	614.8 ± 41.4c	369.8 ± 28.1c
N	345.9 ± 15.9b	29.4 ± 4.70b	247.6 ± 8.89a	83.7 ± 11.5a	706.6 ± 28.6b	504.8 ± 16.9b
NM	397.8 ± 18.6a	34.0 ± 4.26a	259.2 ± 8.34a	74.8 ± 13.1b	765.7 ± 22.7a	556.8 ± 10.7a

AAN: amino acid N, ASN: amino sugar N, AHAN: acid-hydrolysable amino N, AHUN: acid-hydrolysable unidentified N. Values followed by different letters in a column are significantly different at *P* < 0.05. Each column consists of means of triplicated data with ± S.E of means.

### Correlation between *AH*^*15*^*N, NAH*^*15*^*N, AA*^*15*^*N, AHA*^*15*^*N, AS*^*15*^*N, AHU*^*15*^*N* and residual exogenous- ^15^N

The results (Table 5) of correlation coefficient at tillering stage exhibited a positive correlation between residual exogenous ^15^N and *AH*^*15*^*N, NAH*^*15*^*N*, *AHA*^*15*^*N, AS*^*15*^*N* and *AHU*^*15*^*N* (P < 0.01). Also, a negative correlation between *AA*^*15*^*N* and residual exogenous ^*15*^N has been witnessed. At tillering stage, the values of the correlation coefficients with residual exogenous ^*15*^N have been in order as *NAH*^*15*^*N* > *AS*^*15*^*N* > *AHA*^15^*N* > *AH*^*15*^*N* > *AHU*^*15*^*N* with 0.984, 0.982 0.979, 0.800 and 0.631, respectively. Furthermore, a very strong positive correlation has also been observed between *AS*^*15*^*N* and *AHA*^*15*^*N* (0.998), *AS*^*15*^*N* and *NAH*^*15*^*N* (0.960), *AHA*^*15*^*N* and *NAH*^*15*^*N* (0.951). At booting stage, only *NAH*^*15*^*N* has a significant positive correlation (0.888) with residual exogenous-^15^N, whereas *AA*^*15*^*N* has a negative correlation (−0.776) with residual exogenous-^15^N. Moreover, a very strong negative correlation has been established among *AH*^*15*^*N* and *AA*^*15*^*N* (−0.954), *AH*^*15*^*N* and *AHA*^*15*^*N* (−0.899). At maturity stage, residual exogenous-^15^N has significantly positive correlation with the *AH*^*15*^*N, NAH*^*15*^*N, AA*^*15*^*N* and *AS*^*15*^*N* (P < 0.01), while *AS*^*15*^*N* has close correlation with *AH*^*15*^*N, NAH*^*15*^*N* and *AA*^*15*^*N*, (0.980, 0.932 and 0.915, respectively). *AH*^*15*^*N* has also effectively correlated with *NAH*^*15*^*N* and *AA*^*15*^*N* in an order as *AA*^*15*^*N* > *NAH*^*15*^*N* with 0.945 and 0.899 (Table 5).

**Table 5:** Correlation between AH^15^N, NAH^15^N, AA^15^N, AHA^15^N, AS^15^N, AHU^15^N and residual exogenous ^15^N in paddy soil at different growing stages of rice plants under flooded condition (n = 9).

Variables	Residual ^15^N	x_1_	x_2_	x_3_	x_4_	x_5_
**Tillering stage**						
Residual ^15^N	1					
x_1_	0.800^**^	1				
x_2_	0.984^**^	0.714^*^	1			
x_3_	−0.0517 ^ns^	−0.223 ^ns^	−0.0288 ^ns^	1		
x_4_	0.979^**^	0.835^**^	0.951^**^	−0.160 ^ns^	1	
x_5_	0.982^**^	0.834^**^	0.960^**^	−0.127 ^ns^	0.998^**^	1
x_6_	0.631 ^ns^	0.886^**^	0.562 ^ns^	−0.365 ^ns^	0.734^*^	0.736^*^
**Booting stage**						
Residual ^15^N	1					
x_1_	0.607 ^ns^	1				
x_2_	0.888^**^	0.189 ns	1			
x_3_	−0.776^*^	−0.954^**^	−0.422 ^ns^	1		
x_4_	−0.203 ^ns^	−0.899^**^	0.257 ^ns^	0.748^*^	1	
x_5_	−0.314 ^ns^	−0.700^*^	0.00195 ^ns^	0.702^*^	0.702^*^	1
x_6_	−0.100 ^ns^	0.720^*^	−0.533 ^ns^	−0.534 ^ns^	−0.945^**^	−0.677^*^
**Maturing stage**						
Residual ^15^N	1					
x_1_	0.963^**^	1				
x_2_	0.979^**^	0.899^**^	1			
x_3_	0.855^**^	0.954^**^	0.766^*^	1		
x_4_	0.596 ^ns^	0.417 ^ns^	0.690^*^	0.146 ^ns^	1	
x_5_	0.977^**^	0.980^**^	0.932^**^	0.915^**^	0.465 ^ns^	1
x_6_	0.375 ^ns^	0.221 ^ns^	0.429 ^ns^	−0.0477 ^ns^	0.803^**^	0.213 ^ns^

x_1_ represents acid hydrolysable-^15^N (AH^15^N); x_2_ represents non-acidhydrolysible-^15^N (NAH^15^N); x_3_ represents amino acid-^15^N (AA^15^N); x_4_ represents hydrolysible ammonium-^15^N (AHA^15^N); x_5_ represents amino sugar-^15^N (AS^15^N); x_6_ represents hydrolysible unidentified-^15^N (AHU^15^N); “ * and **” indicate significant differences at *P* < 0.05 and *P* < 0.01, respectively, “ns” means no significant difference at *P* < 0.05.

### Urea-^15^N (^15^NU) transformation in the organic nitrogen fractions and their relationships in paddy soil of mono-rice based cropping system

The simple correlation analysis showed that residual ^15^NU was extremely significant and positively correlated with amino sugar nitrogen-^15^N (ASN-^15^N) and acid hydrolysable ammonia nitrogen-^15^N (AHAN-^15^N) in paddy soil at tillering stage (Table 5). Also, it has a correlation with amino acid nitrogen-^15^N (AAN-^15^N), AHAN-^15^N and ASN-^15^N at maturing stage (*P* < 0.01), while negative correlation with AAN-^15^N at booting stage of rice plants (*P* < 0.05). However, the correlation coefficients of the simple correlation analysis usually have a false appearance. Since it comprehensively reflects the direct effect of organic nitrogen component combined with indirect effects of the other components on residual ^15^NU in paddy soil. Hence, in order to determine the effects of organic nitrogen fractions on residual ^15^NU transformation in paddy soil, path analysis has been used. Path analysis identified the components of organic nitrogen that played a direct or indirect role in residual ^15^NU transformation (Table 6).

**Table 6:** Path analysis showing direct and indirect effects of NAH^15^N, AA^15^N, AHA^15^N, AS^15^N and AHU^15^N on residual exogenous-^15^N in paddy soil at different growing stages of rice plants.

Variables	Direct coefficients	Indirect coefficients						
		x_1_	x_2_	x_3_	x_4_	x_5_	x_6_	Total
**Tillering stage**								
x_2_	0.502^**^	0.162	—	−0.001	0.634	−0.225	−0.088	0.482
x_4_	0.667^*^	0.189	0.477	−0.005	—	−0.234	−0.115	0.312
**Booting stage**								
x_1_	0.576^**^	—	0.105	0.163	−0.066	0.009	−0.180	0.031
x_2_	0.555^**^	0.109	—	0.073	0.019	0	0.133	0.334
**Maturing stage**								
x_1_	1.038^**^	—	0.509	−0.520	−0.059	0.017	−0.003	−0.055
x_2_	0.567^**^	0.933	—	−0.426	−0.098	0.017	−0.005	0.420
x_3_	−0.555^*^	0.990	0.434	—	−0.021	0.016	0.001	1.420
x_4_	−0.142 ^ns^	0.433	0.391	−0.081	—	−0.008	−0.009	0.726

x_1_ represents acid hydrolysable-^15^N (AH^15^N); x_2_ represents non-acidhydrolysible-^15^N (NAH^15^N); x_3_ represents amino acid-^15^N (AA^15^N); x_4_ represents hydrolysible ammonium-^15^N (AHA^15^N); x_5_ represents amino sugar-^15^N (AS^15^N); x_6_ represents hydrolysible unidentified-^15^N (AHU^15^N); “ * and **” indicate significant differences at *P *< 0.05 and *P* < 0.01, respectively, “ns” means no significant difference at *P* < 0.05.

Firstly, the optimal stepwise regression equations have been established, in which, *y*_*T*_, *y*_*B*_ and *y*_*M*_ have residual ^15^NU content (mg/kg) in paddy soil at tillering, booting and maturing stage of rice plants, x_1_was non-acid hydrolysable nitrogen-^15^N(NAHN-^15^N) (mg kg^−1^), x_2_ was AAN-^15^N (mg kg^−1^), x_3_ was AHAN-^15^N (mg kg^−1^), x_4_ was ASN-^15^N (mg kg^−1^), x_5_ was acid hydrolysable unidentified nitrogen-^15^N (AHUN-^15^N) (mg kg^−1^). The optimal stepwise regression equations were as follows: *y*_*T*_ = 0.55 + 0.24*x*_1_ + 0.37*x*_3_ (tillering stage), *y*_*B*_ = 0.058 + 0.43*x*_1_ (booting stage), *y*_*M*_ = −0.025 + 0.42*x*_1_−0.56*x*_2_−0.21*x*_3_ (maturing stage). The equations indicated that NAHN-^15^N, AAN-^15^N and AHAN-^15^N mainly affected residual ^15^NU transformation in paddy soil. Furthermore, the correlation coefficients of the equations (r) as mentioned above gradually increased with the gradual introduction of organic nitrogen fractions into equations. That showed the role of the introduced organic nitrogen fractions in the soil on increased residual ^15^NU.

The path analysis compared the direct and indirect effects of organic nitrogen derived from exogenous-^15^NU in different fractions on residual ^15^NU transformation in paddy soil (Table 7). The direct effects of NAHN-^15^N on residual ^15^NU transformation has been extremely significant at each growing stage (*P* < 0.01). Similarly, the direct effect of AHAN-^15^N and AAN-^15^N on residual ^15^NU transformation has been significant at tillering and maturity stage of rice plants, respectively (*P* < 0.05). Furthermore, the indirect effect of AHAN-^15^N through NAHN-^15^N and AAN-^15^N on residual ^15^NU transformation has been greater than its direct effect. Moreover, the indirect coefficient through NAHN-^15^N (0.391) has been the main component of its simple correlation coefficient (0.979). This result illustrated that the newly synthesized AHAN by using exogenous-^15^NU was unstable and may easily convert into the hard degradable organic nitrogen fractions (e.g. NAHN).

**Table 7:** Direct and indirect effects of NAH^15^N, AA^15^N and AHA^15^N on residual ^15^N in paddy soil at different growing stages of rice plants.

Organic N fractions	Direct coefficients of determination	Indirect coefficients of determination			
		x_1_	x_2_	x_3_	Total
**Tillering stage**					
x_1_	0.502^**^	—	−0.001	0.634	0.633
x_3_	0.667^**^	0.477	−0.005	—	0.472
**Booting stage**					
x_1_	0.555^**^	—	0.073	0.019	0.092
**Maturing stage**					
x_1_	0.567^**^	—	−0.426	−0.098	−0.524
x_2_	−0.555^*^	0.434	—	−0.021	0.413
x_3_	−0.142 ^ns^	0.391	−0.081	—	0.310

x_1_ represents acid hydrolysable-^15^N (AH^15^N); x_2_ represents non-acidhydrolysible-^15^N (NAH^15^N); x_3_ represents amino acid-^15^N (AA^15^N); “ * and **” indicate significant differences at *P* < 0.05 and *P* < 0.01, respectively, “ns” means no significant difference at *P* < 0.05.

## Discussion

Certainly, using plant residues instead of synthetic fertilizers will equally boost up yield, other quality traits of the growing crop and maintains quality of the soil with no negative effect on environment. Among various plant nutrients, nitrogen is one of the major elements required by the rice plant during active growth stages^31^. An effective way to fulfill the nitrogen requirements of the rice plants is feeding them with green manure^32^. Milk vetch residues have been proven as one of the best options to nurture the soil with the nutrients that are required by the rice crop^33^. Likewise, the efficient nitrogen enrichment of soil can also be beneficial for the next crop to be grown in a crop rotation system. In this study, we have found high N (both organic and inorganic forms) recovery in the soil from NM plots at all growing stages of rice, where milk vetch residues have added organic-N to the soil. A significantly lower amount of recovered nitrogen in the soil from control pots might be attributed to the zilch addition of the nitrogen from an external source. Similar results have been observed by Rehman *et al*.^34–36^ and Liu *et al*.^37^.

Furthermore, it is quite obvious that the presence of inorganic-N in the soil in high quantities can reflect on high NO_3_^−^–N and NH_4_^+^–N amounts of the soil. This is because of the mineralization process that can take place in the soil by the soil microorganisms. Soil microbes can convert the organic form of nitrogen into more available form, i.e. NH_4_^+^–N, the conversion rate of this process mainly depends on the population density of the microorganisms. Our experiments demonstrated high amounts of NO_3_^−^–N in the soil from N treated pots, which increased during the various growing stages of rice. The presence of high amount of NO_3_^−^–N in N treatments might be due to the application of urea, where remaining NH_4_^+^–N is quickly converted into the NO_3_^−^–N. Low amount of NO_3_^−^–N in NM treated pots might be due to the complex nature of organic-N. Also, zero supplementation of exogenous-N might be the possible explanation for a significantly lower amount of NO_3_^−^–N and NH4^+^–N in CK pots. Besides, we also found a high expression of amoA gene in N treated soil, particularly at the maturity stage of the rice growth. At this particular stage, not only the total count of AOB has been high in N treated pots, but also the aerobic fungi and aerobic Azobacter. The abundance of AOB, aerobic fungi and aerobic Azobacter in the soil at the maturity stage of the rice crop might caused accumulation of NO_3_^−^–N in paddy soil.

The acid hydrolysable-N has been present in higher proportions to non-acid hydrolysable-N in the soil at both tillering and maturity stages, while acid non-hydrolysable-N has been high at booting stage. This difference shows that the acid hydrlysable-N acted as the main component of the N-cycling in the soil at the start and end of the growth stages of the rice plants. On the other hand, non-acid hydrolysable-N served as the main component of the N-cycling in soil at the middle stage. Among the total acid hydrolysable-N, amino acids-N has been the major source of nitrogen for the plants to develop. In addition, AHAN and AHUN have been present in appreciable amounts in the soil at the early growth stages of rice that might have served as a main source of nitrogen. These results are in close agreement with those of Ge *et al*.^38^, Reeve *et al*.^39^ and Chen *et al*.^40^.

The non-acid hydrolysable-N (NAHN), is the main component of humic compounds that are not completely inert and may be degraded and utilized by soil microbes in a short period of time^41^. However, as time passes, most of the exogenous-^15^NU has been reserved in the form of NAHN in paddy soil^42^. Our study also showed that an extremely significant direct effect of NAHN-^15^N on the contribution of the residual ^15^NU transformation in paddy soil during each growing stage of rice plants (Table 7). Obviously, it speculated that the NAHN may be an effective channel of exogenous ^15^NU storage. A portion of AHAN that exists in soil in the forms of fixed ammonium and exchangeable ammonium^43^, can rapidly reserve the exogenous ^15^NU at the early growing stage. The reserved ^15^NU can then be released to meet the nitrogen requirement of rice plants^44^. In addition, the newly synthesized fixed ammonium might be available to rice plants and contributed more to the AHAN-^15^N^45,46^. So, it seemed that the accumulation or release of AHAN-^15^N can be determined by the fixed ammonium-^15^N content in paddy soil.

The indirect effect of AHAN-^15^NthroughNAHN-^15^N and AAN-^15^N on the residual ^15^NU transformation has been greater than its direct effect, which indicated the unstable nature of AHAN-^15^N. This instability of AHAN-^15^N can make it to be assimilated by soil microbes to synthesize its own metabolites with great ease^47^. Besides, it can also be converted into the hard degradable organic nitrogen fractions (e.g. NAHN and ASN), and stored in paddy soil.

Both of the direct and indirect effects of AHAN-^15^N on residual ^15^NU transformation mentioned as above demonstrated that the AHAN maybe a temporary reservoir of the exogenous ^15^NU. This pool of exogenous ^15^NU can further be degraded and released to meet the requirement of rice plants, as soon as it feel for the nitrogen in paddy soil. The relation of absorbed ^15^NU to the AHAN-^15^N content in paddy soil at the filling and harvest stages has been shown previously^42^.

Likewise, in our study, we have opted for efficient use of cultural practices, i.e. fortification of soil with green manure (milk vetch). The accumulation of nitrogen in plant starts from very early stage of plant growth and development, which is evident from our results. Nitrogen from the milk vetch residues (NM treatment) has proved to be the best option regarding accumulation of nitrogen in above-ground parts of rice at all growing stages. Certainly, being a part of enzymes, nitrogen is needed for the normal cell division and elongation during the active growth period of plant life. At the maturity stage, NM treatment played a central role in the accumulation of nitrogen in the straw as well as in filled grains as compared to the N and CK treatments. From these results one can conclude that by fortifying the paddy soil with milk vetch instead of urea can effectively improve the nitrogen dynamics and decrease environmental pollution.

## References

[1] Kearney J (2010). Food consumption trends and drivers. Philosophical transactions of the royal society B: biological sciences.

[2] Delgado JA (2010). Crop residue is a key for sustaining maximum food production and for conservation of our biosphere. Journal of Soil and Water Conservation.

[3] Karlen DL (2009). Crop residues: the rest of the story. Environmental science & technology.

[4] Cruse RM, Herndl CG (2009). Balancing corn stover harvest for biofuels with soil and water conservation. Journal of Soil and Water Conservation.

[5] Delgado JA, Dillon MA, Sparks RT, Essah SY (2007). A decade of advances in cover crops. Journal of Soil and Water Conservation.

[6] Delgado J, Del Grosso S, Ogle S (2010). 15N Isotopic crop residue cycling studies suggest that IPCC methodologies to assess N2O-N emissions should be reevaluated. Nutrient Cycling in Agroecosystems.

[7] Kümmerer K, Held M, Pimentel D (2010). Sustainable use of soils and time. Journal of soil and water conservation.

[8] Constantin J (2010). Effects of catch crops, no till and reduced nitrogen fertilization on nitrogen leaching and balance in three long-term experiments. Agriculture, ecosystems & environment.

[9] Poeplau C, Don A (2015). Carbon sequestration in agricultural soils via cultivation of cover crops–A meta-analysis. Agriculture, Ecosystems & Environment.

[10] Nascente AS, Crusciol CAC, Cobucci T (2013). The no-tillage system and cover crops—Alternatives to increase upland rice yields. European Journal of Agronomy.

[11] Godfray HCJ (2010). Food security: the challenge of feeding 9 billion people. science.

[12] Mohanty M (2011). Modelling N mineralization from green manure and farmyard manure from a laboratory incubation study. Ecological Modelling.

[13] Ling-ling LI, Shu-tian LI (2014). Nitrogen Mineralization from Animal Manures and Its Relation to Organic N Fractions. Journal of Integrative Agriculture.

[14] Tejada M, Gonzalez J, García-Martínez A, Parrado J (2008). Application of a green manure and green manure composted with beet vinasse on soil restoration: effects on soil properties. Bioresource technology.

[15] Yang Z (2011). Effects of long-term winter planting-green manure on microbial properties and enzyme activities in reddish paddy soil. Soils.

[16] Vanlauwe B, Zingore S (2011). Integrated soil fertility management: an operational definition and consequences for implementation and dissemination. Better Crops.

[17] Schleper C (2010). Ammonia oxidation: different niches for bacteria and archaea?. Isme Journal.

[18] Norton, J. M. Diversity and environmental distribution of ammonia-oxidizing bacteria. *Nitrification. ASM Press, Washington, DC*, 39–55 (2011).

[19] Ai C (2013). Different roles of rhizosphere effect and long-term fertilization in the activity and community structure of ammonia oxidizers in a calcareous fluvo-aquic soil. Soil Biology and Biochemistry.

[20] Jiang Y, Jin C, Sun B (2014). Soil aggregate stratification of nematodes and ammonia oxidizers affects nitrification in an acid soil. Environmental microbiology.

[21] Page, A. *Methods of soil analysis: chemical and microbiological proerpteis*. (Amen Society of Agronomy, 1982).

[22] Hoagland, D. R. & Arnon, D. I. The water-culture method for growing plants without soil. *Circular. California Agricultural Experiment Station* **347** (1950).

[23] Buresh R, Austin E, Craswell E (1982). Analytical methods in 15 N research. Nutrient Cycling in Agroecosystems.

[24] Stevenson, F. Nitrogen-organic forms. *Methods of Soil Analysis Part 3—Chemical Methods*, 1185–1200 (1996).

[25] Wu Y (2009). Effects of different soil weights, storage times and extraction methods on soil phospholipid fatty acid analyses. Geoderma.

[26] Wu Y (2009). Changes in the soil microbial community structure with latitude in eastern China, based on phospholipid fatty acid analysis. Applied Soil Ecology.

[27] Bååth E, Anderson T-H (2003). Comparison of soil fungal/bacterial ratios in a pH gradient using physiological and PLFA-based techniques. Soil Biology and Biochemistry.

[28] Frostegård Å, Bååth E (1996). The use of phospholipid fatty acid analysis to estimate bacterial and fungal biomass in soil. Biology and Fertility of Soils.

[29] Zelles L (1997). Phospholipid fatty acid profiles in selected members of soil microbial communities. Chemosphere.

[30] Dewey DR, Lu K (1959). A correlation and path-coefficient analysis of components of crested wheatgrass seed production. Agronomy journal.

[31] Mei-Hua D (2012). Optimizing nitrogen fertilizer application for rice production in the Taihu Lake region, China. Pedosphere.

[32] Zhou C (2016). Integration of growing milk vetch in winter and reducing nitrogen fertilizer application can improve rice yield in double-rice cropping system. Rice Science.

[33] Fageria N (2007). Green manuring in crop production. Journal of Plant Nutrition.

[34] Rahman MM, Amano T, Shiraiwa T (2009). Nitrogen use efficiency and recovery from N fertilizer under rice-based cropping systems. Australian Journal of Crop Science.

[35] Rahman M, Islam M, Azirun M, Boyce A (2014). Agronomic and nitrogen recovery efficiency of rice under tropical conditions as affected by nitrogen fertilizer and legume crop rotation. Plant Sci.

[36] Rahman, M. M., Islam, A. M., Azirun, S. M. & Boyce, A. N. Tropical legume crop rotation and nitrogen fertilizer effects on agronomic and nitrogen efficiency of rice. *The Scientific World Journal* **2014** (2014). 10.1155/2014/490841 PMC4055622 24971378

[37] Liu X (2005). Crop production, nitrogen recovery and water use efficiency in rice–wheat rotation as affected by non-flooded mulching cultivation (NFMC). Nutrient Cycling in Agroecosystems.

[38] Ge T (2009). Amino acids as a nitrogen source for tomato seedlings: the use of dual-labeled (13 C, 15 N) glycine to test for direct uptake by tomato seedlings. Environmental and Experimental Botany.

[39] Reeve JR, Smith JL, Carpenter-Boggs L, Reganold JP (2009). Glycine, nitrate, and ammonium uptake by classic and modern wheat varieties in a short-term microcosm study. Biology and fertility of soils.

[40] Chen X-y, Wu L-h, Cao X-c, Zhu Y-h (2013). Organic nitrogen components in soils from southeast China. Journal of Zhejiang University Science B.

[41] Schulten H-R, Schnitzer M (1997). The chemistry of soil organic nitrogen: a review. Biology and Fertility of Soils.

[42] Jiang H (2014). Transformation of external chemical nitrogen in soil organic nitrogen fractions and their relationship. Journal of Plant Nutrition and Fertilizer.

[43] Stevenson, F. J. *Humus chemistry: genesis, composition, reactions*. (John Wiley & Sons, 1994).

[44] Drury C, Voroney R, Beauchamp E (1991). Availability of NH 4+-N to microorganisms and the soil internal N cycle. Soil Biology and Biochemistry.

[45] Zhang Y-Z (2007). Fixed ammonium content and maximum capacity of ammonium fixation in major types of tillage soils in Hunan province, China. Agricultural Sciences in China.

[46] Lu C (2010). Fixation of labeled (15 NH 4) 2 SO 4 and its subsequent release in black soil of Northeast China over consecutive crop cultivation. Soil and Tillage Research.

[47] He H, Lü H, Zhang W, Hou S, Zhang X (2011). A liquid chromatographic/mass spectrometric method to evaluate 13C and 15N incorporation into soil amino acids. Journal of Soils and Sediments.

