# Author Correction: The role of Chinese Milk Vetch as cover crop in complex soil nitrogen dynamics in rice rotation system of South China

**DOI:** 10.1038/s41598-018-38074-5

**Published:** 2019-04-03

**Authors:** Zhijian Xie, Chunhuo Zhou, Farooq Shah, Amjad Iqbal, GuoRong Ni

**Affiliations:** 1 College of Land Resource and Environment, Jiangxi Agricultural University, Nanchang Jiangxi, 330045 P.R. China; 2 Department of Agriculture, Abdul Wali Khan University Mardan, 23200 Kyber Pakhtunkhwa, Pakistan

Correction to: *Scientific Reports* 10.1038/s41598-018-30239-6, published online 13 August 2018

This Article contains typographical errors in the Acknowledgements section.

“and the Special Funds for Agro-Scientific Research in the Public Interest (201503123).”

should read:

“and the Special Funds for Agro-Scientific Research in the Public Interest (201303106).”

